# Effects of the application of a checklist during trauma resuscitations on ATLS adherence, team performance, and patient-related outcomes: a systematic review

**DOI:** 10.1007/s00068-019-01181-7

**Published:** 2019-08-07

**Authors:** Oscar E. C. van Maarseveen, Wietske H. W. Ham, Nils L. M. van de Ven, Tim F. F. Saris, Luke P. H. Leenen

**Affiliations:** 1 Department of Trauma Surgery, University Medical Center Utrecht, Heidelberglaan 100, 3584 CX Utrecht, The Netherlands; 2 Institute of Nursing Studies, University of Applied Sciences Utrecht, Heidelberglaan 7, 3584 CS Utrecht, The Netherlands

**Keywords:** Trauma resuscitation, Checklist, Adherence, Process- and patient related outcome

## Abstract

**Purpose:**

In this systematic literature review, the effects of the application of a checklist during in hospital resuscitation of trauma patients on adherence to the ATLS guidelines, trauma team performance, and patient-related outcomes were integrated.

**Methods:**

A systematic review was performed following the Preferred Reporting Items for Systematic Reviews and Meta-analyses checklist. The search was performed in Pubmed, Embase, CINAHL, and Cochrane inception till January 2019. Randomized controlled- or controlled before-and-after study design were included. All other forms of observational study designs, reviews, case series or case reports, animal studies, and simulation studies were excluded. The Effective Public Health Practice Project Quality Assessment Tool was applied to assess the methodological quality of the included studies.

**Results:**

Three of the 625 identified articles were included, which all used a before-and-after study design. Two studies showed that Advanced Trauma Life Support (ATLS)-related tasks are significantly more frequently performed when a checklist was applied during resuscitation. [14 of 30 tasks (*p* < 0.05), respectively, 18 of 19 tasks (*p* < 0.05)]. One study showed that time to task completion (− 9 s, 95% CI = − 13.8 to − 4.8 s) and workflow improved, which was analyzed as model fitness (0.90 vs 0.96; *p* < 0.001); conformance frequency (26.1% vs 77.6%; *p* < 0.001); and frequency of unique workflow traces (31.7% vs 19.1%; *p* = 0.005). One study showed that the incidence of pneumonia was higher in the group where a checklist was applied [adjusted odds ratio (aOR) 1.69, 95% Confidence Interval (CI 1.03–2.80)]. No difference was found for nine other assessed complications or missed injuries. Reduced mortality rates were found in the most severely injured patient group (Injury Severity score > 25, aOR 0.51, 95% CI 0.30–0.89).

**Conclusions:**

The application of a checklist may improve ATLS adherence and workflow during trauma resuscitation. Current literature is insufficient to truly define the effect of the application of a checklist during trauma resuscitation on patient-related outcomes, although one study showed promising results as an improved chance of survival for the most severely injured patients was found.

## Background

A specialized trauma team should resuscitate severely injured trauma patients. The goal was to identify and treat life-threatening injuries at an early stage. Previous studies have shown that severely injured patients resuscitated by a trauma team have a higher chance of survival compared to trauma patients resuscitated without a trauma team [1–4]. Specialized trauma teams are composed of a variable number of medical specialists and paramedical workers and typically consist of a trauma surgeon or an emergency physician, an anesthetist, a radiologist, in some cases a neurologist, emergency nurses, and a diagnostic radiographer. To reduce the time from injury to critical interventions, a predefined set of consecutive tasks is performed by the trauma team members [5].

In most cases, available information about the health status of severely injured patients during resuscitation is limited, while there are potentially many different injuries that could be life-threatening. To adequately identify and treat potentially life-threatening injuries, a full-scale and structured assessment is required. The Advanced Trauma Life Support^™^ (ATLS^™^) guidelines are accepted worldwide and are the structural standard for the systematic resuscitation of trauma patients [6]. The ATLS^™^ guidelines provide guidance to the trauma team during trauma resuscitation by prioritizing diagnostic and treatment procedures.

Previous studies reported that tasks recommended by the ATLS^™^ guidelines are not always performed during trauma resuscitation, whereas adherence to ATLS^™^ guidelines varied between 42 and 82% [7–11]. Although procedures within the ATLS™ guidelines are constructed in a chronological and coherent manner, human factors may challenge the natural limitations of our memory [12, 13]. A previous study at our institute illustrates that 35 tasks should be performed during the first 7 min, as recommended by the ATLS^™^ [14]. Since there are many tasks to fulfill in limited time, tasks could easily be overlooked. Thereby, common situations during trauma resuscitation such as stressful circumstances, sudden interruptions and lack of experience of trauma team members affect the capacity of memory and, therefore, may contribute to suboptimal adherence of ATLS^™^ guidelines [15–18].

A checklist could complement the naturally limited human memory, which could be even more impaired by the stress felt during initial resuscitation. In general, a checklist could be used as a reminder, in order to completely and adequately fulfill a single complex procedure or a sequence of procedures. For example, safety checklists have been shown to improve medical care and have been used in medicine for several years. Thomassen et al. [19] performed a systematic review in 2014 and summarized medical literature to show the effects of safety checklist. Thirty-four studies were included, of which some had investigated well known-safety checklists such as the SURgical PAtient Safety System (SURPASS) or the World Health Organization (WHO) surgical safety checklist. The auteurs concluded that the use of safety checklist had reduced mortality and morbidity in targeted patients groups [19]. In addition, safety checklists have strengthened compliance with guidelines, improved human factors, and reduced the incidence of adverse events [19]. Comparable positive effects were found by a more recent studies, of Ramsay et al. [20] who investigated the introduction of the SURPASS in Scotland and of de Jager et al. [21] who performed a single center observational study, investigating the effect of the introduction of the surgical safety checklist with a follow-up time of 5 years.

The capability of checklists to complement human memory may also improve trauma resuscitation. Therefore, we aimed to systematically review the literature to determine the effects of the application of a checklist during trauma resuscitation of a trauma patient by a trauma team on adherence to the ATLS™ guidelines, trauma team performance, and patient-related outcomes.

## Methods

The systematic review adhered to the preferred reporting items for systematic reviews and meta-analyses (PRISMA) [22].

### Search strategy

A systematic search of studies listed in the electronic databases of Pubmed, Embase, CINAHL, and Cochrane was performed from their inception till January 2019. We combined the search terms derived from our research aim in separate search strings for each database (Table 1). Our search terms covered the intended population (Trauma resuscitation, including accessory search terms) and intervention (the application of a checklist, including accessory search terms). By purpose, we did not use search terms for outcomes, in order to avoid the introduction selection bias, as we intended to research all possible outcomes. In addition, all reference lists of included articles were screened for relevant additional citations.

**Table 1:** Each database-specific search term used to identify articles concerning subject matter

Database	Search terms
Pubmed	(“Checklist”[Mesh] OR checklist*[tiab] OR check list*[tiab]) AND (“Wounds and Injuries”[Mesh] OR Trauma[tiab]) AND (Primary Survey[tiab] OR Secondary Survey[tiab] OR resuscitation*[tiab] OR “Resuscitation”[Mesh] OR team*[tiab])
Embase	‘checklist’/exp OR checklist*:ab,ti OR (check NEXT/1 list*):ab,ti AND (‘injury’/exp OR trauma:ab,ti) AND (‘primary survey’:ab,ti OR ‘secondary survey’:ab,ti OR resuscitation*:ab, ti OR ‘resuscitation’/exp OR team*:ab,ti)
CINAHL	((MH “Checklists”) OR checklist* OR check list*) AND ((MH “Trauma”) OR (MH “Wounds and Injuries”) OR trauma) AND (Primary Survey OR Secondary Survey OR resuscitation* OR (MH “Resuscitation”) OR team*)
Cochrane	(checklist* OR check list*) AND (Trauma) AND (Primary Survey OR Secondary Survey OR resuscitation* OR team*) Limited on; Title, abstract, keywords

### Eligibility criteria

Studies were included if the effect of the application of a checklist during resuscitation of trauma patients by a trauma team in an in-hospital setting was evaluated. We included studies with a randomized controlled—or controlled before-and-after study design. All other forms of observational study designs, reviews, case series or case reports, animal studies, and simulation studies were excluded. Solely English and Dutch publications from peer-review journals were reviewed. There were no restrictions on the year of publication, on the age of patients or on composition or method of application of a checklist during trauma resuscitation.

### Study selection

First, all eligible studies were selected by screening the title and abstract. Second, all selected papers were screened full-text. During these two phases, two investigators independently selected articles according to the predefined eligibility criteria and discussed their results. In case of any unresolved disagreements, a third reviewer made the final decision using the same eligibility criteria. The resultant articles after the two phases of screening were included in our systematic review.

### Data collection and critical appraisal

Two reviewers independently extracted and inserted the following data into tables: Authors, year of publication, study design, research population, checklist’s items, composition, form, and application during trauma resuscitation and all identified effects reported by the included articles. To assess the methodological quality of the included studies, the Effective Public Health Practice Project Quality Assessment Tool (EPHPP tool) was applied [23]. This tool has been judged suitable to be used in systematic reviews of effectiveness [24] and has been reported to have content and construct validity [25, 26]. Furthermore, this tool is a reliable instrument to assess the quality of randomized controlled trials and before-and-after studies, whereas the overall inter-observer coefficient was found to be 0.77 (95% CI 0.51–0.90) [27]. This inter-observer coefficient value is considered as an excellent agreement [28] (Table 2).

**Table 2:** Overview of included studies: used checklist, effect measured, main results and study quality

Auteurs	Study design/sites/population/method	Checklist items/composition/form/application	Effects measured	Main results	Global EHPPH rating
Kelleher et al. [29]	Controlled before-and-after study	30 ATLS items	Adherence	Fourteen of the 30 ATLS tasks were completed more often after checklist introduction (for all *p* ≤ 0.01)	Moderate
	Monocenter	Local Delphi procedure			
				After adjustment the odds were 2.66 (95% CI 2.07–3.42) and 2.46 (95% CI 2.04–2.98) times higher for completing primary survey tasks, respectively, secondary survey after the introduction of a checklist	
	435 pediatric trauma patients	Paper checklist	Time to task completion	Vital sign measurements were obtained faster (*p* ≤ 0.01 for all) after the checklist was implemented	
	Retrospective analysis of video taped trauma resuscitations	Surgical or emergency medicine physician team leader			
				After adjustment primary survey tasks were performed faster (*p* < 0.001) after the checklist was implemented	
Kelleher et al. [30]	Controlled before-and-after study	30 ATLS items	Workflow	After checklist implementation, the fitness (0.80 vs 0.91; *p* = 0.007) and conformance (26.1% vs 59.4%; *p* = 0.01) improved for resuscitations without notification	Moderate
	Monocenter	Local Delphi procedure			
	435 pediatric trauma patients	Paper checklist			
	Retrospective analysis of video taped trauma resuscitations	Surgical or emergency medicine physician team leader			
Lashoher et al. [31]	Controlled before-and-after study	19 core items of which 11 are ATLS items	Adherence	18 of the 19 tasks clinical tasks were significant (*p* < 0.05) more frequently performed after implementation of the WHO trauma care checklist	Weak
	Multicenter, 11 sites	Based on literature review of medical errors during initial resuscitation of severely injured patients	Complications	The incidence of one of the ten complications (pneumonia) was slightly higher after the introduction of the checklist (AOR 1.69, 95% CI 1.03–2.80). There AOR for the other nine complications was not significantly different	
	3422 adult trauma patients	Not described	Missed injuries	Incidence of missed injuries did not differ before and implementation of a checklist (AOR 0.62; 95% CI 0.19–2.03; *p* = 0.437)	
	Trauma resuscitation was assessed by live observants	Not described	Mortality	No difference in odds of mortality in the overall study sample (OR 1.02; CI 0.77–1.34 *p* = 0.904	
				50% reduction (AOR 0.51; 95% CI 0.30–0.89; *p* = 0.018) in mortality among patients with the most severe injuries (ISS > 25)	

## Results

### Selection

**Fig. 1:**
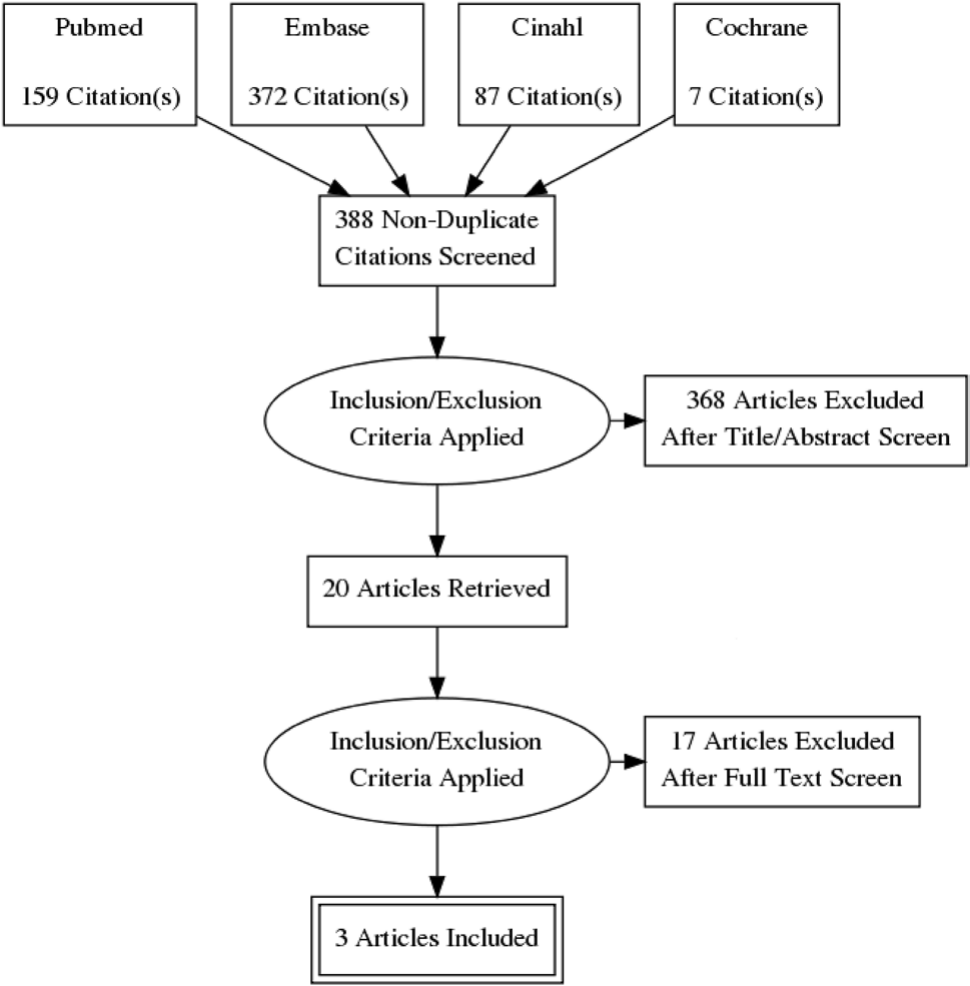
Study attrition diagram

The search strategy yielded 625 potentially relevant titles and abstract. After screening title and abstract, 20 articles remained for full-text screening (Fig. 1). After screening the full texts of remaining articles, three studies were included in our systematic review. No additional studies were found by assessing the reference lists of included studies.

### Study characteristics

All three included studies had a before-and-after study design to investigate the effect of the application of a checklist during trauma resuscitation. Two included studies of Kelleher et al. [29, 30] originated from the same pediatric trauma center and had the same sample. Two separate analyses and corresponding results were published in two different articles [30]. In their studies all trauma resuscitations had been captured on video during two 4-month periods and were analyzed retrospectively. They included 435 and 437 cases in their analyses, respectively. The third included study, of Lashoher et al. [31], was a multicenter study, including 11 hospitals in eight different countries. A random sample of 20% of all trauma resuscitations was assessed by live observants. Data collection duration varied among the different hospitals and ranged from 3 to 8 months pre- and 3–11 months post-implementation. In total, 3422 resuscitations had been assessed and the results were analyzed.

### Critical appraisal

Using the EPHPP quality assessment tool, the quality of the two studies of Kelleher et al. [29, 30] and Lashoher et al. [31] were found to be moderate and weak, respectively (Table 3). Most concerning point that affected the quality of all three studies was the awareness of the participants (trauma team members) of being studied. This effect, also known as the Hawthorne effect [32], could have influenced behavior during trauma resuscitations. Two other points of concern were found in the study of Lashoher et al. [31], whereas no description of the randomization, withdrawal, and dropout of trauma resuscitations was provided.

**Table 3:** Critical appraisal following he Effective Public Health Practice Project Quality Assessment Tool

Authors	Selection bias	Study design	Confounders	Blinding	Data collection method	Withdrawals and dropouts	Global rating
Kelleher et al. [29]	Moderate	Moderate	Strong	Weak	Moderate	Not applicable	Moderate
Kelleher et al. [30]	Moderate	Moderate	Strong	Weak	Moderate	Not applicable	Moderate
Lashoher et al. [31]	Weak	Moderate	Strong	Weak	Moderate	Weak	Weak

### The checklists

Two different checklists were applied in the three included studies. A paper checklist was used in the two studies of Kelleher et al. [28, 29]. The checklist was applied by the surgical team leader (surgical senior resident, fellow, or attending) or a member of the emergency medicine leadership team (emergency medicine fellow or attending) during trauma resuscitation. The checklist was composed of 30 items extracted from the ATLS^™^ guidelines, of which 14 items belong to the primary survey and 16 items belong to the secondary survey. The checklist was designed to track task completion by the bedside clinician (surgical junior resident or trauma nurse practitioner) and other team members.

In the study of Lashoher et al. [31], the application of the World Health Organization Trauma Care Checklist was investigated. The WHO trauma care Checklist is based on a literature review of medical errors during initial resuscitation of severely injured patients [33], and contains 19 core items, of which 11 items are tasks also described in the ATLS^™^ guidelines. Hospital staff of all 11 hospitals participating in the study received checklist- and patient safety training.  Furthermore, hospital staff of each participating hopsital were encouraged to modify the checklist to be relevant in their unique setting. The study team of the core checklist approved site-specific final versions. The responsible member of the trauma team and form (e.g. paper or electronic) of the checklist during resuscitation was not described in the included article.

### Effects measured

Six different outcomes-related to the applications of a checklist during trauma resuscitation were identified: Mortality, complications, missed injuries, task adherence, mean time to task completion, and workflow.

### Mortality

Lashoher et al. [31] found no difference in odds of mortality in the overall study sample after adjusting for patient demographics and injury severity. After an injury-severity-stratified analysis, checklist implementation was associated with a 50% reduction (adjusted odds ratio (aOR) 0.51, 95% Confidence Interval (CI 0.30–0.89) in mortality among patients with the most severe injuries (Injury severity score > 25). No such association was found among patients who were less severely injured (injury severity score < 25) [31].

### Complications

Only the incidence of one of the ten complications, pneumonia, was slightly higher after the introduction of the checklist after adjusting for patient characteristics (aOR 1.69, 95% CI 1.03–2.80) in the study of Lashoher et al. [31]. No differences were found for the other nine assessed complications [31].

### Missed injuries

The incidence of missed injuries was zero in both groups and did not differ before and after implementation of a checklist after adjusting for patient characteristics and injury severity (aOR 0.62; 95% CI 0.19–2.03; *p* = 0.437) in the study of Lashoher et al. [31].

### Task adherence

Fourteen of the thirty ATLS-related tasks that had been assessed in the study of Kelleher et al. [29] were completed significantly more frequently after checklist introduction (for all *p* ≤ 0.05). None of the 30 ATLS-related were performed less frequently. After adjustment for type of resuscitation factors and type of tasks (tasks were not further specified), the odds of completing primary survey tasks were 2.66 (95% CI 2.07–3.42) times higher after implementation, and the adjusted odds of secondary survey task completion were 2.46 (95% CI 2.04–2.98) times higher. Lashohor et al. [31] found 18 of the 19 clinical tasks to be significantly (*p* < 0.05) more frequently performed after implementation of the WHO trauma care checklist.

### Mean time to task completion

Kelleher et al. [30] found that the average adjusted time to task completion was 9 s faster with the application of a checklist (95% CI − 13.8 to − 4.8 s) Furthermore, six of the 12 primary survey tasks (full exposure, temperature measurement, blood pressure, heart rate measurement, respiratory rate measurement, and oxygen saturation) were performed significantly (all *p* < 0.05) faster (− 20 till − 43 s) [29].

### Workflow

A predefined ideal process model of six ATLS primary survey tasks was created in the study of Kelleher et al. [30] The six ATLS tasks were categorized into Airway (A), Breathing (B), Circulation (C), and Disability (D) which is well known from the ABCDE mnemonic as described in the ATLS Guidelines. The model required that “A” tasks are done before “B” tasks, “B” tasks are done before “C” tasks, and “C” tasks are done before “D” task. Model fitness (degree of agreement with the ideal process model, ranging from 0 to 1), conformance (“yes” if fitness 1; “no” if fitness < 1) was measured and the frequency of unique workflow traces were analyzed. Besides the comparison of the application of a checklist, analysis of a brief pre-arrival notification was performed. The authors found that after implementation of a checklist workflow improved. Model fitness (0.80 vs 0.91; *p* = 0.007), conformance frequency (26.1% vs 59.4%; *p* = 0.01), and number of unique workflow traces (19 vs 16; *p* = 0.01) improved for resuscitations without notification. Comparable improvement was shown after the implementation of the checklist when a brief notification was given, in which case Model fitness (0.90 vs 0.96; *p* < 0.001), conformance frequency (50.8% vs 77.6%; *p* < 0.001), and frequency of unique workflow traces (63 vs 35; *p* = 0.005) also improved. Finally, the effect of the absence of pre-arrival notification on model fitness was also smaller after checklist implementation compared to before the use of the checklist (Cohen’s *d* = 0.75 (95% CI 0.73–0.82) vs. Cohen’s *d* = 0.52 (95% CI 0.51–0.56).

## Discussion

Our systematic review shows that the application of a checklist may improve adherence to ATLS guidelines and workflow during resuscitation. Furthermore, we found that the application of a checklist might lead to a reduction of mortality among severely injured patients. However, there was no effect found on missed injuries and the incidence of one out of ten complications investigated was even slightly higher when a checklist was applied during resuscitation.

Several limitations in our systematic review and included studies should be considered. First, only three studies could be included and besides adherence to task performance, all effects including patient-related outcomes were investigated only in one study per effect. Therefore, no meta-analysis or other forms of analysis were performed on the collected data.

Second, the quality of the only study investigated patient-related outcome was weak [31]. The limitations in this study may have affected the effect of a checklist on patient-related outcomes. For example, the increased incidence of pneumonia in patients resuscitated with the application of a checklist is unexpected. The authors clarified that there may be systematic errors or misclassifications in both process and outcome measures, such as different available imaging modalities across centers for the diagnosis of complications such as pneumonia. Furthermore, the incidence of missed injuries was zero in both groups. Previous studies, however, show that missed injuries vary between 1.3 and 39% [34]. These low numbers may, therefore, be a result of information bias, which was caused by suboptimal registration of missed injuries. Furthermore, the study of Lashoher et al. [31] did not only measure the effect of a checklist on trauma resuscitation. Hospital staff received checklist- and patient safety training in the context of trauma care and, therefore, the impact of checklist implementation was also studied.

Third, the populations of two of the three studies were pediatric trauma patients. Although ATLS principles are used for resuscitation of injured children and adult patients, interpretation of cardiorespiratory variables, airway anatomy, response to blood loss, thermoregulation, and the trauma team composition are different in pediatric resuscitation and may have influenced ATLS adherence rates and workflow [35, 36].

Finally, different checklists were applied in the included studies. We did not predefine the type and application style of the checklist for trauma resuscitation since we did not find any evidence in literature on optimal composition and application of checklists during trauma resuscitation. The composition of the checklists was different in the included studies, whereas 30 items were included in the checklist applied in the Kelleher studies and in the study of Lashoher et al. [31] 14 tasks with variation per hospital were formed based on a literature search.

Although this systematic review and included articles have several limitations, the application of a checklist during trauma resuscitation may improve patient- and process- related outcomes. The improvement of ATLS adherence is in line with the results of studies investigating the application of checklists in other fields of medicine, whereas adherence to guidelines for surgical safety procedures and adherence to protocols improved [20, 37–42].

Additional research is needed for a more reliable estimation of the effect of the application of a checklist during trauma resuscitation on patient’s- and process-related outcomes. Future studies investigating the effect of the application of a checklist should use a before-and-after study design, whereas in a randomized controlled design introduces recall bias as the members of the trauma team not using the checklist are likely to remember details about the checklist. To minimize bias by observers, the same observer(s) should assess the trauma resuscitations. Analysing videos of recorded trauma resuscitations could obviate the need for multiple observers to observe all resuscitations, especially during the night and weekend, or when there are multiple trauma resuscitations at the same time. Furthermore, missed injuries should be actively searched, for example, by a 30-day follow-up with a clinical re-evaluation of included patients and a retrospective re-evaluation of performed diagnostic imaging. Finally, to our knowledge, no research has been performed to investigate optimal checklist composition and application for trauma resuscitation. Improvement of composition and application may improve the user-friendliness of a checklist and may potentially improve the trauma resuscitation process even further.

Although the evidence is marginal, the application of a checklist during trauma resuscitation seems to improve processes during trauma resuscitation and may even improve the chance to survive for the most severely injured patients. Thereby, no adverse events were noticed at the three included studies. On the other hand, the implementation of a checklist may be paired with several barriers. In the study of Nolan et al. [43] barriers to implementing the World Health Organization’s Trauma Care Checklist are described, including unclear roles, lack of enforcement, poor understanding of the purpose and professional hierarchy. These barriers were comparable to barriers that have been described by previous studies investigating the implementing of the WHO’s Surgical Safety Checklist [44–47], which have shown to reduce mortality and morbidity [20, 48].

In conclusion, considering the results of our systematic review including limitations and included studies, the application of a checklist could be useful and may be considered to be introduced into daily practice. Further research is needed for a more reliable estimation of the effect of the application of a checklist during trauma resuscitation on patient- and process-related outcomes.
